# Supplementary figures and images for: Specificity for deubiquitination of monoubiquitinated FANCD2 is driven by the N-terminus of USP1
Source: Life Sci Alliance. 2018 Oct 12;1(5):e201800162. doi: 10.26508/lsa.201800162 (PMC6238601; doi:10.26508/lsa.201800162)

**Figure 1C**

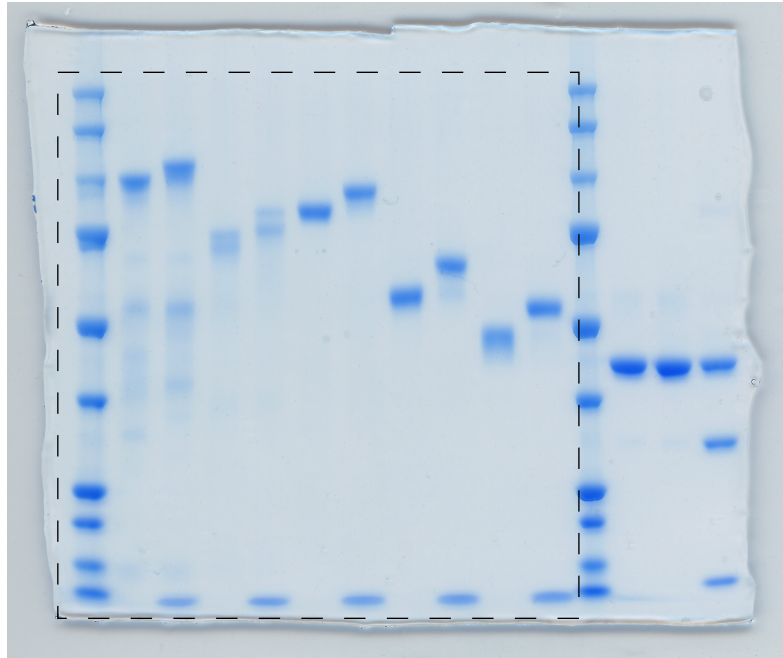

**Figure 1F**

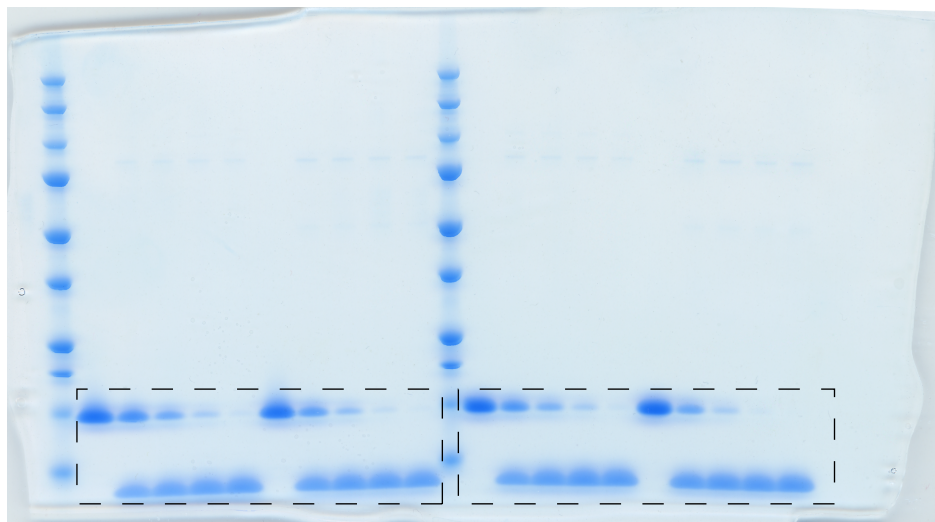

K63-diubiquitin

K48-diubiquitin

Supplement: Supplementary file 1 [file LSA-2018-00162_SdataF1.pdf]

**Figure 3A**

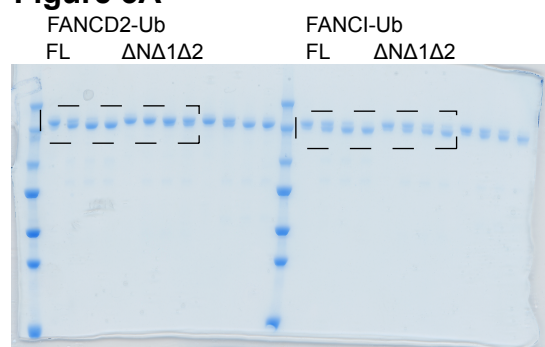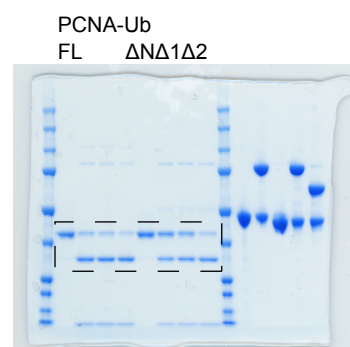

**Figure 3B**

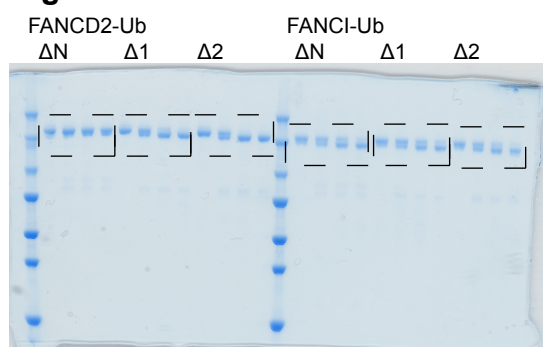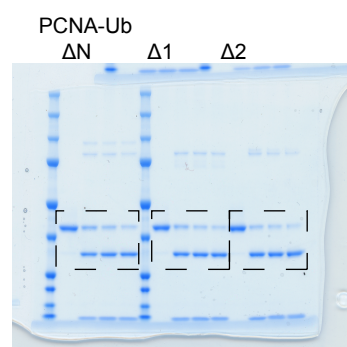

**Figure 3C**

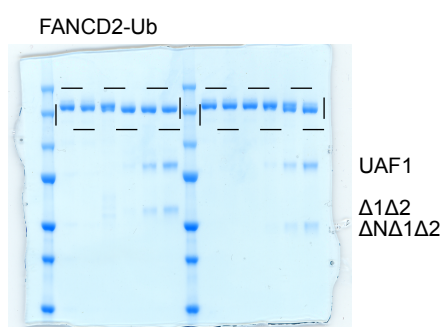

**Figure 3D**

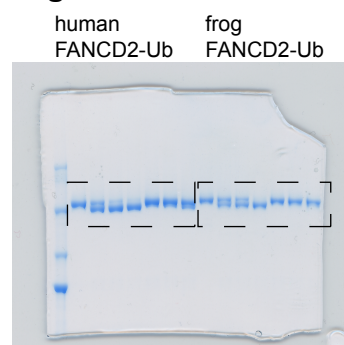

Supplement: Supplementary file 3 [file LSA-2018-00162_SdataF3.pdf]

**Figure 4B**

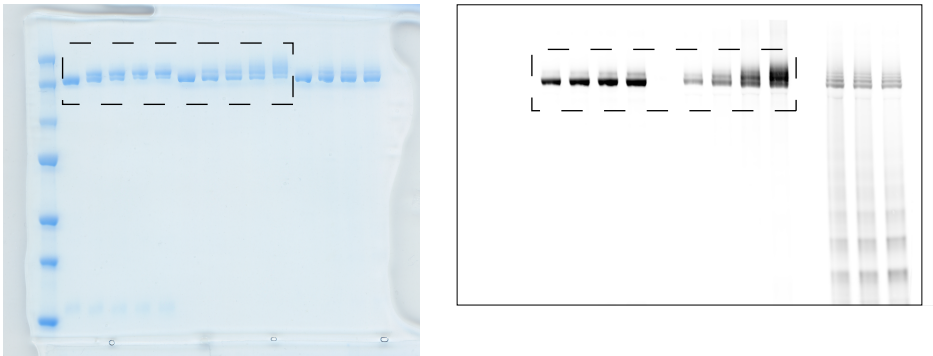

**Figure 4C**

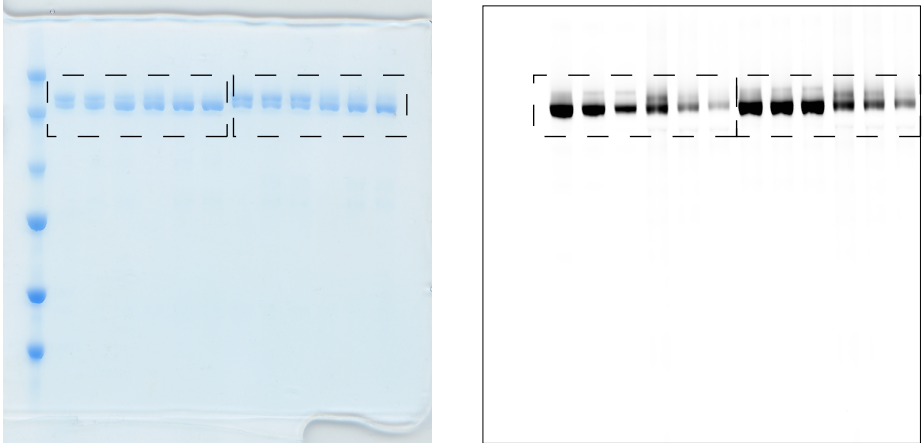

Supplement: Supplementary file 4 [file LSA-2018-00162_SdataF4.pdf]

**Figure 5B**

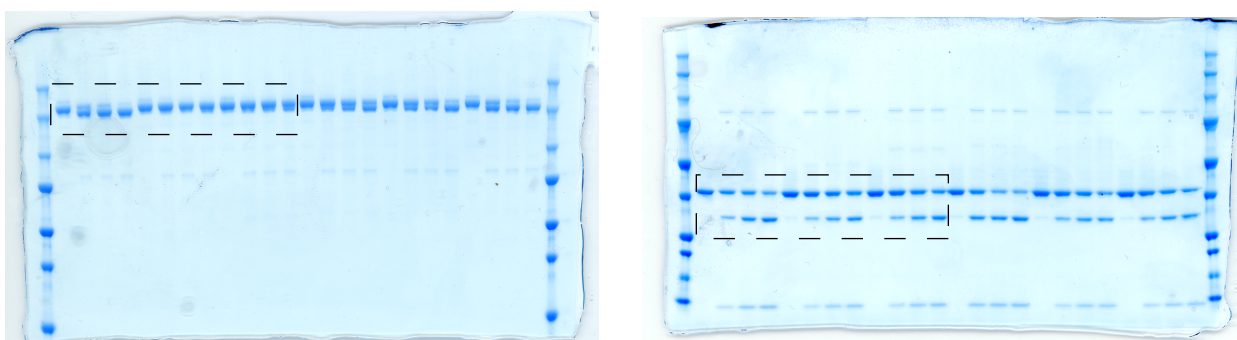

**Figure 5C**

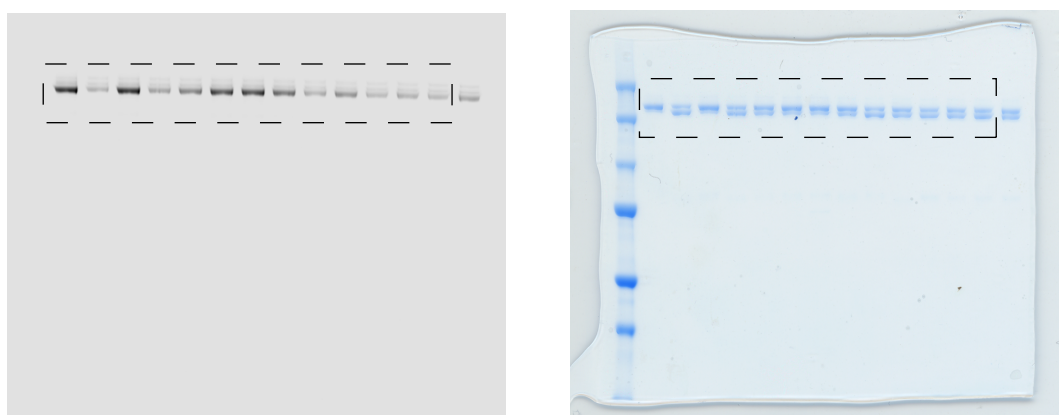

**Figure 5D**

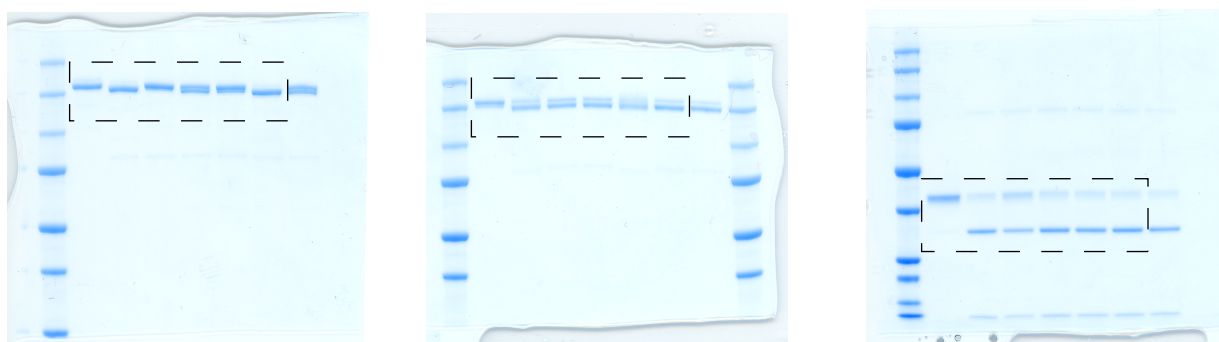

Supplement: Supplementary file 5 [file LSA-2018-00162_SdataF5.pdf]

**Figure 7A**

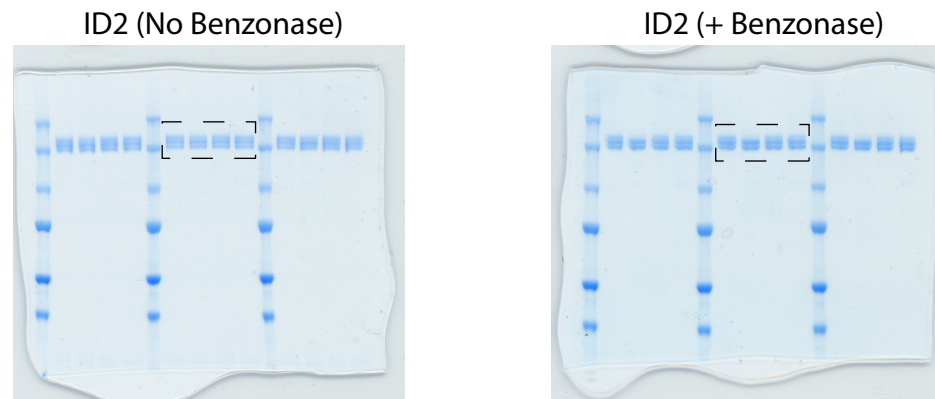

**Figure 7B**

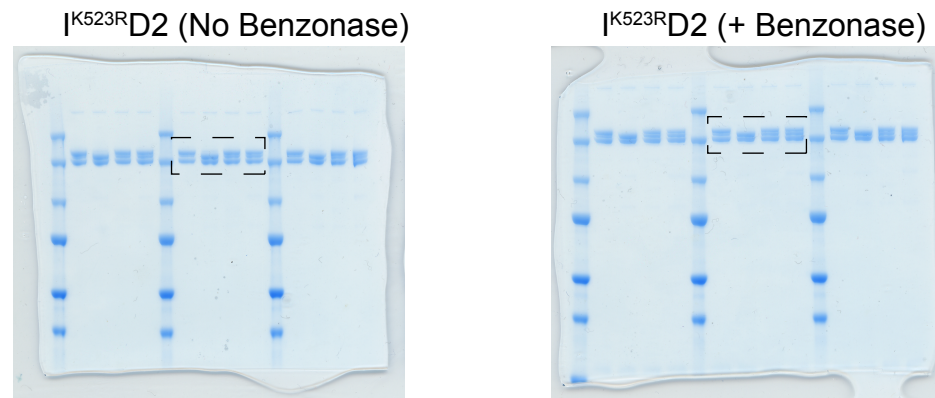

**Figure 7A and 7B**

Western blots - all on same membrane  
green = FANCD2 and red = FANCI

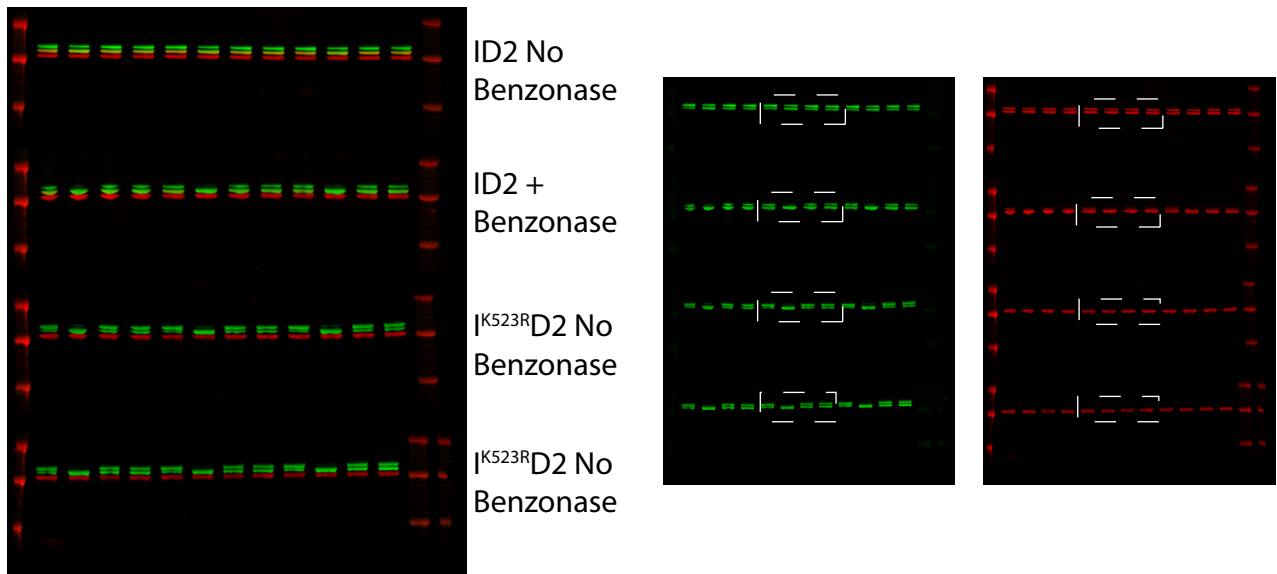

Supplement: Supplementary file 7 [file LSA-2018-00162_SdataF7.pdf]
