# Supplementary material for: Specificity for deubiquitination of monoubiquitinated FANCD2 is driven by the N-terminus of USP1
Source: Life Sci Alliance. 2018 Oct 12;1(5):e201800162. doi: 10.26508/lsa.201800162 (PMC6238601; doi:10.26508/lsa.201800162)
Supplement: Supplementary file 2 [file LSA-2018-00162_SdataF2.pdf]

Figure 2A

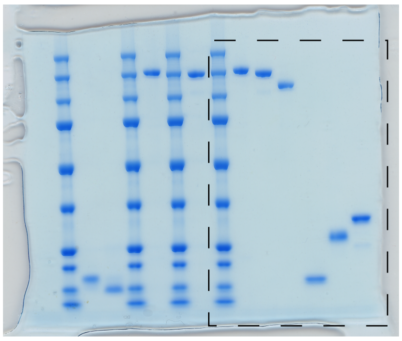

Figure 2B

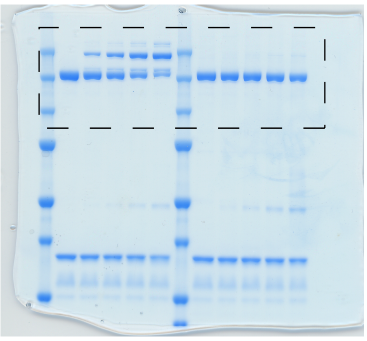

FANCD2-Ub

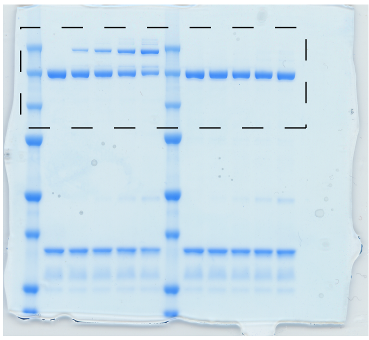

FANCI-Ub

Figure 2C

FANCI-Ub

FANCD2-Ub

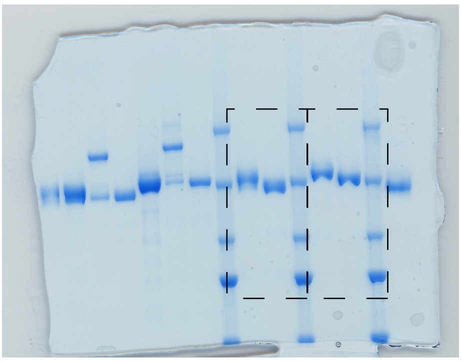

PCNA-Ub

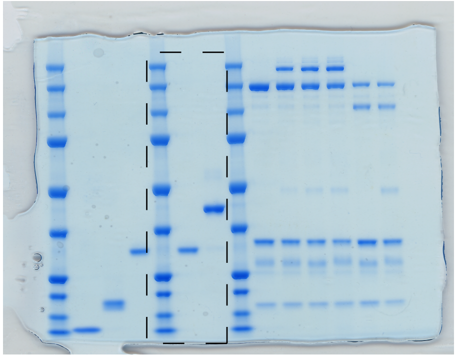

Figure 2D

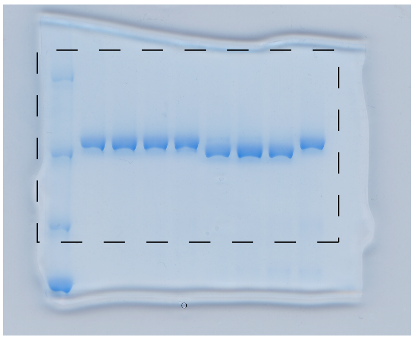

FANCD2-Ub

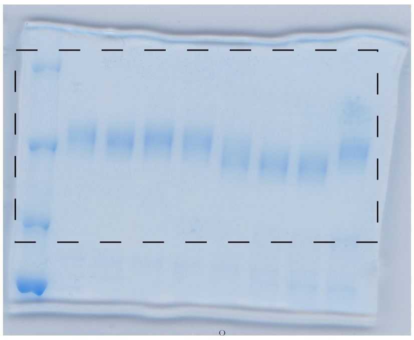

FANCI-Ub

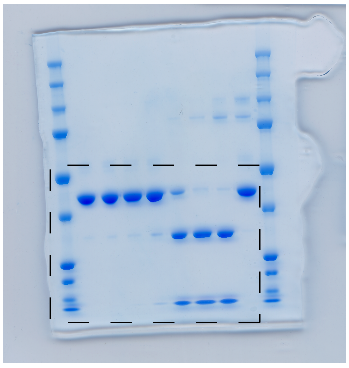

PCNA-Ub
