# Supplementary material for: Specificity for deubiquitination of monoubiquitinated FANCD2 is driven by the N-terminus of USP1
Source: Life Sci Alliance. 2018 Oct 12;1(5):e201800162. doi: 10.26508/lsa.201800162 (PMC6238601; doi:10.26508/lsa.201800162)

Figure 6A

USP1$^{\Delta1\Delta2}$ activity on FANCD2-Ub
USP1$^{\Delta N\Delta1\Delta2}$ activity on FANCD2-Ub

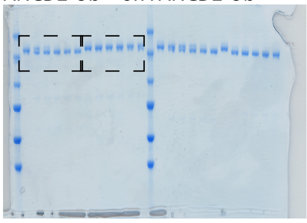

USP2 activity on FANCD2-Ub

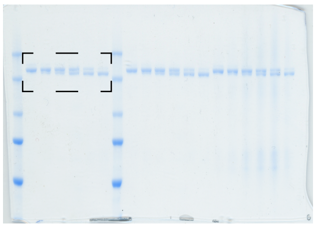

USP1-USP2 activity on FANCD2-Ub

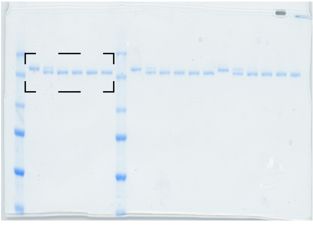

Figure 6B

USP1$^{\Delta1\Delta2}$ activity on FANCI-Ub
USP1$^{\Delta N\Delta1\Delta2}$ activity on FANCI-Ub

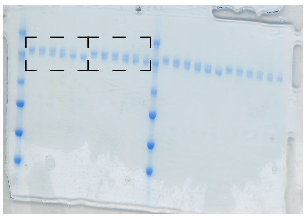

USP2 activity on FANCI-Ub

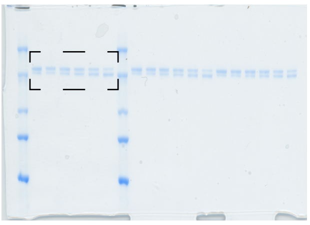

USP1-USP2 activity on FANCI-Ub

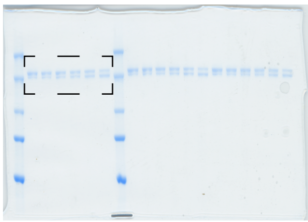

Figure 6C

USP1$^{\Delta1\Delta2}$ activity on PCNA-Ub
USP1$^{\Delta N\Delta1\Delta2}$ activity on FANCI-Ub

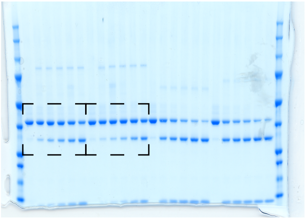

USP2 activity on PCNA-Ub

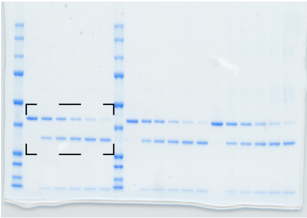

USP1-USP2 activity on PCNA-Ub

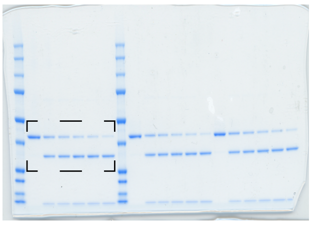

Supplement: Supplementary file 6 [file LSA-2018-00162_SdataF6.pdf]
